# Guanine Can Direct Binding Specificity of Ru–dipyridophenazine (dppz) Complexes to DNA through Steric Effects

**DOI:** 10.1002/chem.201605508

**Published:** 2017-02-06

**Authors:** James P. Hall, Sarah P. Gurung, Jessica Henle, Patrick Poidl, Johanna Andersson, Per Lincoln, Graeme Winter, Thomas Sorensen, David J. Cardin, John A. Brazier, Christine J. Cardin

**Affiliations:** ^1^ Department of ChemistryUniversity of Reading, WhiteknightsReadingRG6 6ADUK; ^2^ Diamond Light Source, Harwell Science and Innovation CampusFermi AvenueDidcotOX11 0QXUK; ^3^ Department of Chemistry and Chemical EngineeringChalmers University of Technology412-96GothenbergSweden; ^4^ Department of Chemistry - BMCUppsala University, Box 576751 23UppsalaSweden; ^5^ Department of PharmacyUniversity of Reading, WhiteknightsReadingRG6 6ADUK

**Keywords:** DNA, DNA structures, nucleic acids, structural biology

## Abstract

X‐ray crystal structures of three Λ‐[Ru(L)_2_dppz]^2+^ complexes (dppz=dipyridophenazine; L=1,10‐phenanthroline (phen), 2,2′‐bipyridine (bpy)) bound to d((5BrC)GGC/GCCG) showed the compounds intercalated at a 5′‐CG‐3′ step. The compounds bind through canted intercalation, with the binding angle determined by the guanine NH_2_ group, in contrast to symmetrical intercalation previously observed at 5′‐TA‐3′ sites. This result suggests that canted intercalation is preferred at 5′‐CG‐3′ sites even though the site itself is symmetrical, and we hypothesise that symmetrical intercalation in a 5′‐CG‐3′ step could give rise to a longer luminescence lifetime than canted intercalation.

Octahedral ruthenium complexes have been studied since the mid‐1980s owing to their ability to interact with DNA.^1^ Ru–dipyridophenazine (dppz) complexes are of particular interest, because they are able to act as DNA probes^2^ or can induce guanine oxidation^3^ when exposed to light. Their photophysical properties can be sensitive to the binding site, in which they are bound, in particular with Δ‐[Ru(bpy)_2_(dppz)]^2+^ showing strong and diagnostic emission when bound to DNA mismatch sites,^4^ with information about the binding specificity indispensable for data interpretation.

Both [Ru(phen)_2_(dppz)]^2+^ and [Ru(bpy)_2_(dppz)]^2+^ can act as luminescent DNA probes and display different emission lifetimes when bound to different types of DNA, and even with a homogeneous DNA polymer, such as (poly(dA‐dT))_2_ and (poly(dG‐dC))_2_, two emission lifetimes were observed.^5^ Barton and co‐workers proposed that the multiple lifetimes arise from different binding geometries,^6^ and recently it was suggested that long emission lifetimes are associated with a canted binding mode, with short lifetimes due to a symmetrical binding motif when bound to AT‐DNA.5c, 7

Both [Ru(phen)_2_(dppz)]^2+^ and [Ru(bpy)_2_(dppz)]^2+^ are DNA light‐switch compounds,^8^ which demonstrate luminescence in non‐polar environments, although emission is turned off in aqueous medium. This has been reported to be because hydrogen bonding between the pyrazine nitrogen atoms and water allows the complex to revert to the ground state through a dark state.^9^ This quenching process is proportional to the number of hydrogen bonds between solvent molecules and the dppz pyrazine nitrogen atoms, and as such, emission is sensitive to how the complex is bound at its binding site. We have reported that the Λ enantiomer of [Ru‐ (phen)_2_(dppz)]^2+^ can bind to 5′‐CC‐3′,^10^ 5′‐TC‐3′^11^ and 5′‐TG‐3′^12^ steps by canted intercalation. In this binding mode, the dppz group is canted towards one side of the DNA, protecting one nitrogen from the solvent. However, when the same complex binds into a 5′‐TA‐3′ step, it adopts a symmetrical binding mode, intercalating deeply and with a high twist angle, causing both dppz pyrazine nitrogen atoms to be accessible to solvent. Thus, there is structural data that supports the hypothesis that long and short emission lifetimes arise from a canted and a symmetrical binding mode, respectively, but understanding both the preferences for different binding sites and bound geometry is key to interpreting measurements performed in solution.^13^

To date, this symmetrical binding mode has only been observed at a 5′‐TA‐3′ step. Symmetrical binding to a 5′‐AT‐3′ step is clearly disfavoured which has been shown for Λ‐[Ru‐ (phen)_2_(dppz)]^2+^ by X‐ray crystallography and, for the isostructural Λ‐[Ru(TAP)_2_(dppz)]^2+^, through ultrafast transient spectroscopic experiments in solution.^13^

This presents only two other possible steps, in which symmetrical intercalation could occur: 5′‐CG‐3′ and 5′‐GC‐3′. However, intercalation to a purine/pyrimidine step is known to be far less common,^14^ possibly because the stacking free energy of 5′‐GC‐3′ is more stabilising to the duplex than 5′‐CG‐3′.^15^ Herein, we present three X‐ray crystal structures containing Λ‐Ru–dppz complexes bound to a 5′‐CG‐3′ step by canted intercalation (Figure 1). These structures reveal precisely how binding is directed by a G NH_2_ group to give a canted binding mode, altering the orientation of the complex when bound into a 5′‐CG‐3′ or 5′‐GC‐3 ’′step. We also extrapolate a hypothetical 5′‐CG‐3′ symmetrical binding site from this model and show how this would be different from binding at a 5′‐TA‐3′ step.

**Figure 1:**
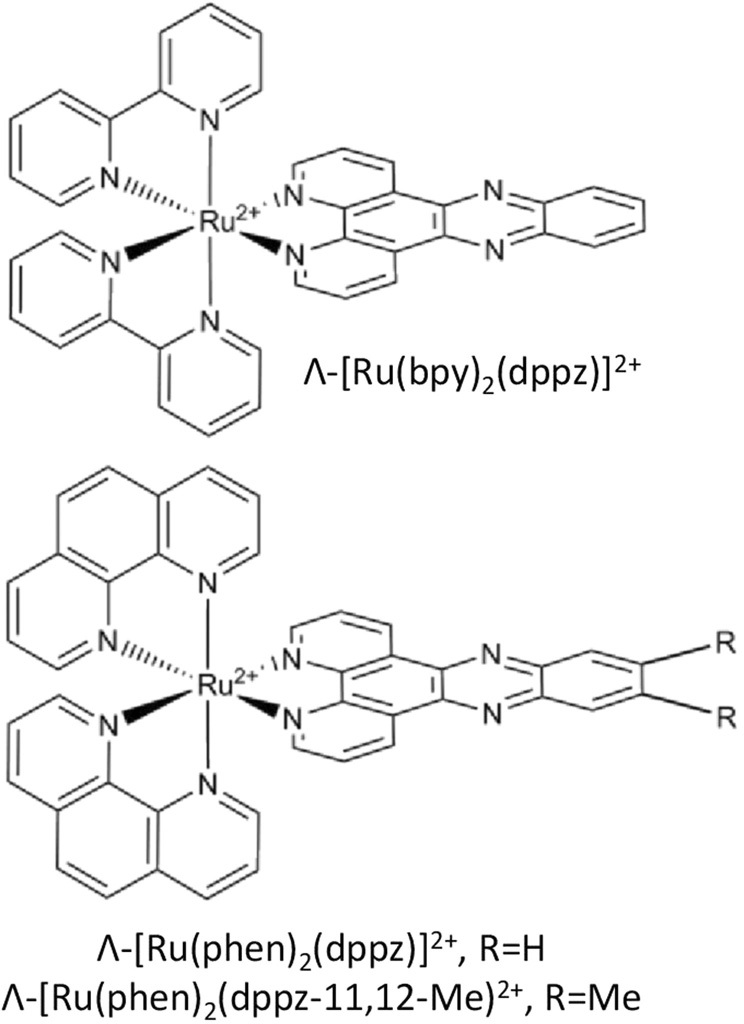
Schematic diagram of the three complexes used in this study.

The structures contain Λ‐[Ru(bpy)_2_(dppz)]^2+^ (**1**), Λ‐[Ru‐ (phen)_2_(dppz)]^2+^ (**2**) and Λ‐[Ru(phen)_2_(dppz‐11,12‐Me)]^2+^ (**3**) bound to a non‐self‐complementary DNA duplex of sequence d((5‐BrC)GGC) (strand 1) with d(GCCG) (strand 2). In all three structures, the complex intercalates into the 5′‐(5‐BrCG)‐3′ step with a canted binding mode (Figure 2 a–c). At the binding site, the long axis of the dppz group is offset from the base hydrogen bonds by 44° on one side and 90° on the other and the complex is intercalated from the minor groove. The twist angle at the intercalation site is 28°, a reduction of 8° compared to standard B‐DNA. The overall conformation of the DNA, as was assigned by the sugar puckering, is that of an unwound B‐DNA. The ancillary ligands of the complex pack onto symmetry‐related equivalent groups of a complex in a neighbouring asymmetric unit (Figure S3 in the Supporting Information).

**Figure 2:**
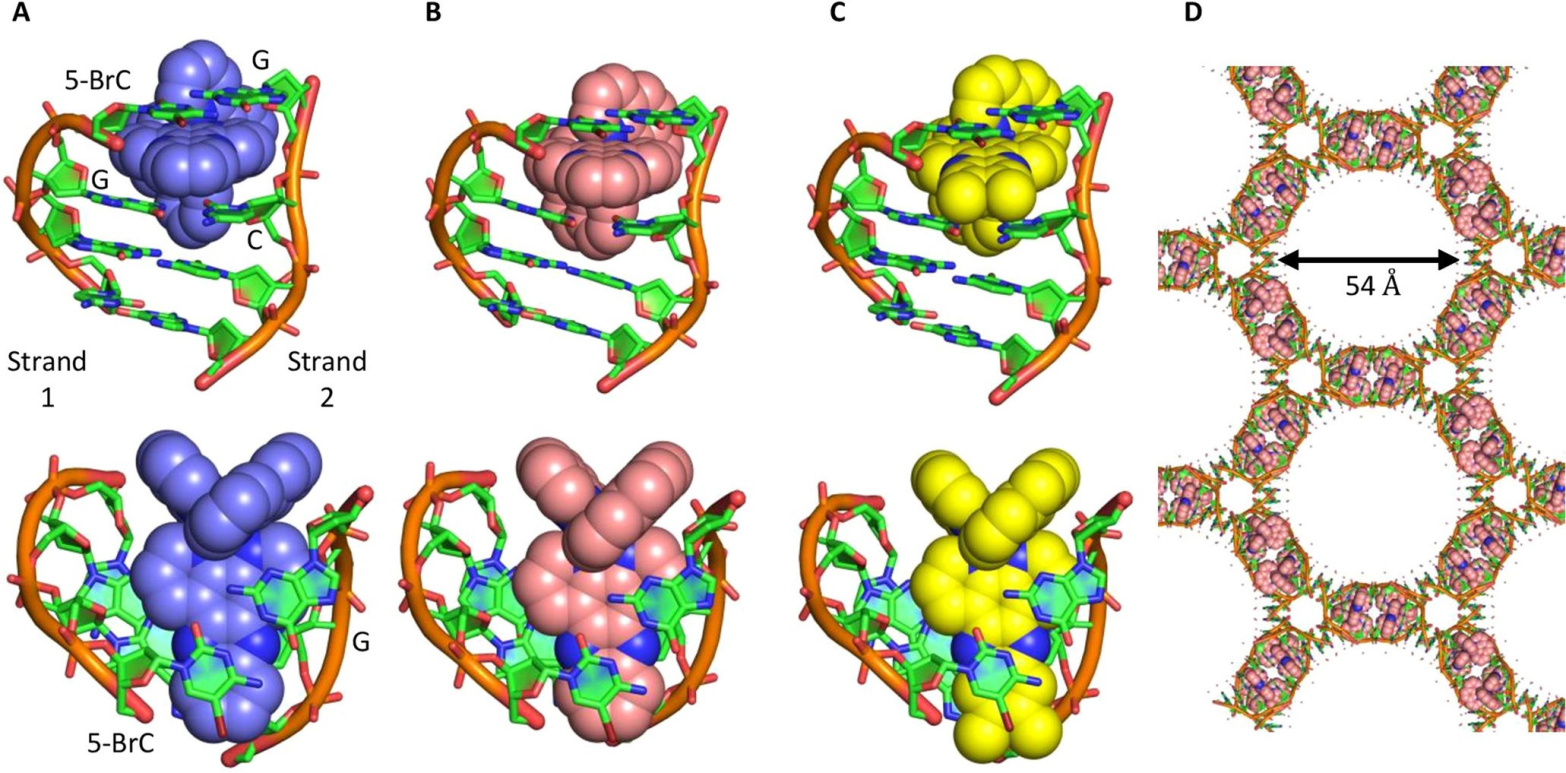
Three X‐ray crystal structures of an octahedral Ru–dppz complex bound to d((5Br‐C)GGC).(GCCG) by canted intercalation. A) Structure 1: Λ‐[Ru‐ (bpy)_2_(dppz)]^2+^ (light blue); B) structure 2: Λ‐[Ru(phen)_2_(dppz)]^2+^ (pink); C) structure 3: Λ‐[Ru(phen)_2_(dppz‐11,12‐Me)]^2+^ (yellow); D) crystal packing, viewed down the *c* axis, forms large solvent channels 54 Å wide. DNA atoms are coloured according to type with carbon in green, nitrogen in blue, phosphorus in orange, oxygen in red and bromine in brown.

The crystals have a high solvent content (72 %) and contain 54 Å wide solvent channels, which run through the length of the crystal in the *c* direction (Figure 2 d). At the binding site, the dppz group stacks predominantly onto the cytosine bases, and the complex does not intercalate deeply enough for the dppz group to be located underneath the Br in the substituted 5‐BrC. Intriguingly, whilst attempts were made to crystallize the same system with non‐brominated DNA, crystals, in both related and different conditions, were not obtained. Using an analogous brominated DNA‐8mer, it was observed that bromination of the DNA increases its stability (Figure S4 in the Supporting Information). Therefore, we ascribe the ability of the 4‐mer sequence to crystallize with the Λ‐ruthenium complex to be a combination of stabilisation by the brominated base and intercalation of the complex, which is known to increase the *T*_m_.^16^

The structure of the binding site and DNA is virtually identical in structures **1**–**3** and, therefore, they will be discussed as a single entity from now on. However, the fact that they are so similar shows that there is little difference in binding between bpy and phen‐based Λ complexes (Figure S2 in the Supporting Information) and that methyl‐substitution at the 11th and 12th positions on the dppz does not change how the compounds bind, which is consistent with the crystal structure^17^ of Λ‐[Ru(TAP)_2_(dppz‐11,12‐Me)]^2+^ bound to d(TCGGCGCCGA)_2_. This observation shows that, as long as no major structural changes are made, Λ‐Ru‐dppz complexes can be treated as a single class of compound when it comes to their binding specificity. Luminescent life‐times, binding enthalpy and nearest neighbour cooperativity parameters are very similar for Λ‐[Ru(phen)_2_(dppz)]^2+^ and Λ‐[Ru(bpy)_2_(dppz)]^2+^ binding to [poly(dA‐dT)]_2_. In strong contrast, the corresponding Δ enantiomers show differences between the phen and bpy analogues.^7^

In all three structures, the complex is bound with one ancillary group (bpy/phen) packed against G_4_ on strand 2, resting against the backbone and hydrogen atom on the (G_4_)2 NH_2_ group (Figure 3 a and b). A consequence of binding is that between C_3_ and G_4_ on strand 2, the backbone elongates with a *γ* torsion angle of 153° instead of its standard angle of approximately 60°, creating an asymmetric cavity. The second ancillary group packs against (strand 1) G_2_ and (strand 2) C_3_. The ancillary group sits on the C side of the minor groove, in a cavity formed between the (strand 1) (G_2_)2 NH_2_ and (strand 2) C_3_ (Figure 3 a). The dppz group sits directly above the C−G hydrogen bonds and is not directed towards either base. Previously, we reported an X‐ray structure of both enantiomers of [Ru‐ (phen)_2_(dppz)]^2+^ simultaneously bound to the TG/CA steps in d(ATGCAT)_2_.^12^ The Λ enantiomer was bound with a different orientation to the Δ, possibly as a consequence of significant unwinding of the DNA duplex, which is present outside of the two base pairs adjacent to the binding site. At the Δ binding site, one phen moiety packs against the DNA backbone, which has a similar *γ* torsion angle to that found in structures **1**–**3** and similarly asymmetric cavity. The second phen packs against (strand A) G_3_, and fits into a pocket formed by the 2‐NH_2_ group and the G_3_ sugar (Figure 3 c and d). In this case, the dppz group is directed toward the G side of the duplex and centrally located between the two bases, in contrast to that observed in structures **1**–**3**. It is again the hydrogen atom on the G_2_ NH_2_ group, directed into the DNA minor groove that forms this cavity, differentiating adenine from guanine. The orientation of the dppz is maintained with both enantiomers, and is a direct consequence of the G_2_ NH_2_ group blocking the ancillary ligands from the complex from sitting at an equal distance between the two phosphate groups at the intercalation site. This observation provides a structural rationale why symmetrical intercalation is expected to be favoured at 5′‐TA‐3′ sites, and also illustrates that the binding geometry at a site, with a guanine base, would be determined by the steric hindrance of the G_2_ NH_2_ group. In contrast, luminescence lifetime studies have consistently reported the presence of two species in solution for Λ‐[Ru(phen)_2_(dppz)]^2+^ bound to [poly(dG‐dC)]. It is possible that some symmetrical intercalation could be present in solution, but that small differences in the binding mode differentiate this from symmetrical binding at a 5′‐TA‐3′ step. To address this point, a hypothetical model was constructed by using a previously reported structure as a starting point.^10^ The bases were changed to 5′‐CG‐3′ and the bound molecule was reoriented to minimise steric clashes.

**Figure 3:**
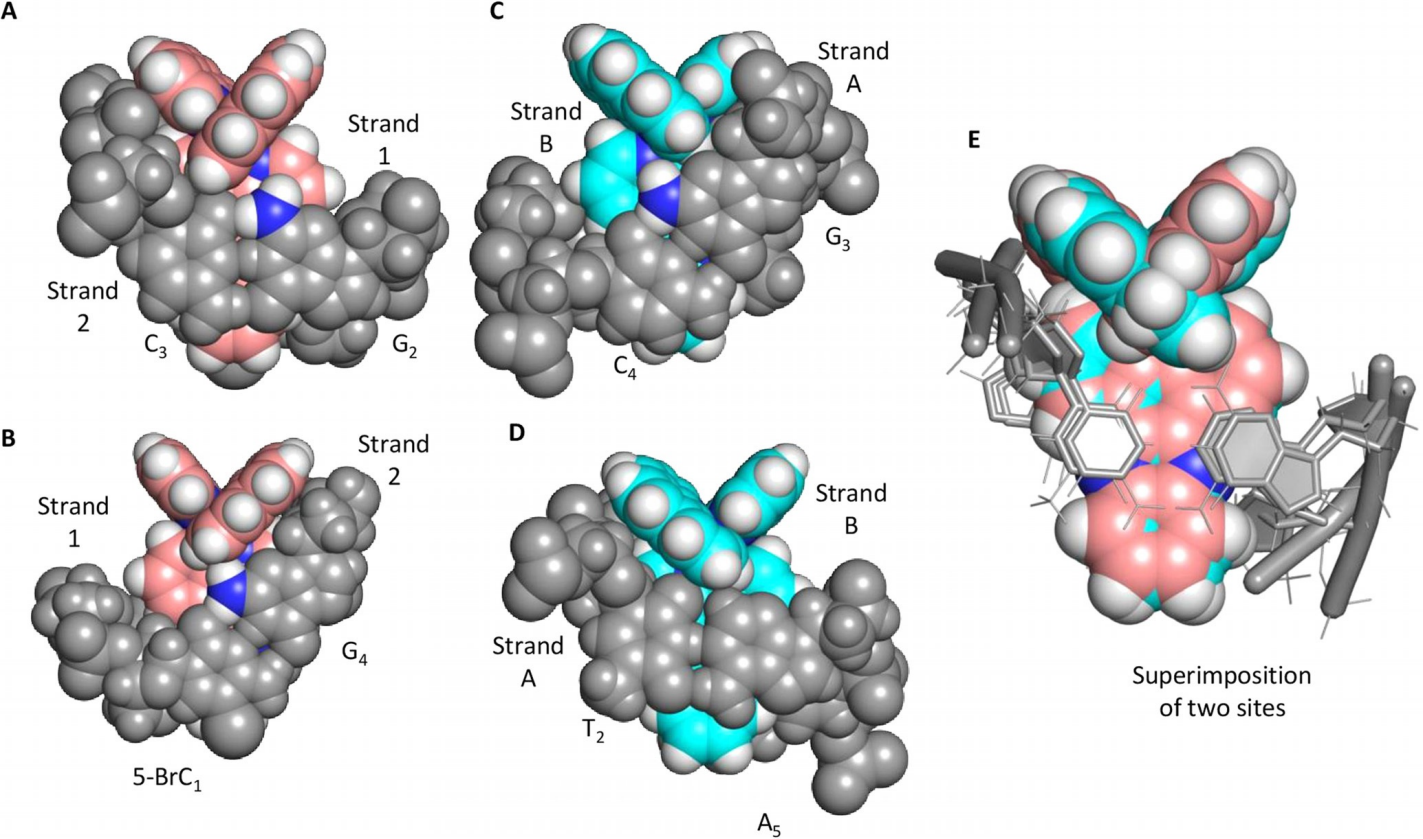
A, B) Two views of the binding site for Λ‐[Ru(phen)_2_(dppz)]^2+^ (pink spheres) bound into the 5′‐(5‐BrC)G‐3′ step of d((5Br‐C)GGC).(GCCG). In A, the phen group is adjacent to the G NH_2_ (blue and white). Please note that this is only formed on one side of the duplex DNA. In B, the phen packs against G_4_. C, D) Δ‐[Ru(phen)_2_(dppz)]^2+^ (cyan spheres) bound into a 5′‐TG‐3′ step in d(ATGCAT)_2_.^11^ C) In this site, a binding pocket is again formed by the guanine 2 NH_2_ group (blue and white spheres). D) As in A, the second phen group packs against a base (T_2_). E) Superimposition of the two sites. All DNA atoms, apart from the guanine NH_2_ group, are displayed in grey. For the G NH_2_, nitrogen atoms are presented in blue and hydrogen is in white.

This model shows that the complex would bind symmetrically at the DNA intercalation site with a depth of intercalation, which would place the dppz nitrogen atoms under the DNA bases (Figure 4). As a result of this, these atoms would have reduced accessibility to solvent molecules, in contrast to symmetric binding at a 5′‐TA‐3′ step. At this step, the dppz penetrates the DNA more deeply (Figure S5 in the Supporting Information), to the extent that the dppz nitrogen atoms are able to interact with the solvent in the major groove. Symmetric binding at a 5′‐CG‐3′ step could therefore result in a longer emission lifetime than for the canted mode, because the phenazine nitrogen atoms seem to be better protected from solvent, and this would offer a more protected site than those observed for any of the binding sites with AT‐rich DNA. Therefore, this is the opposite assignment of solvent accessibility to that proposed for symmetrical intercalation into TA/TA steps, which we also propose to be the preferred binding site of Λ enantiomers in mixed sequence DNA. Quenching of emission by guanine has been previously reported,5c which may explain why only a relatively short lifetime was observed in solution when Λ‐[Ru(phen)_2_(dppz)]^2+^ is bound to [poly(dG‐dC)].5c

**Figure 4:**
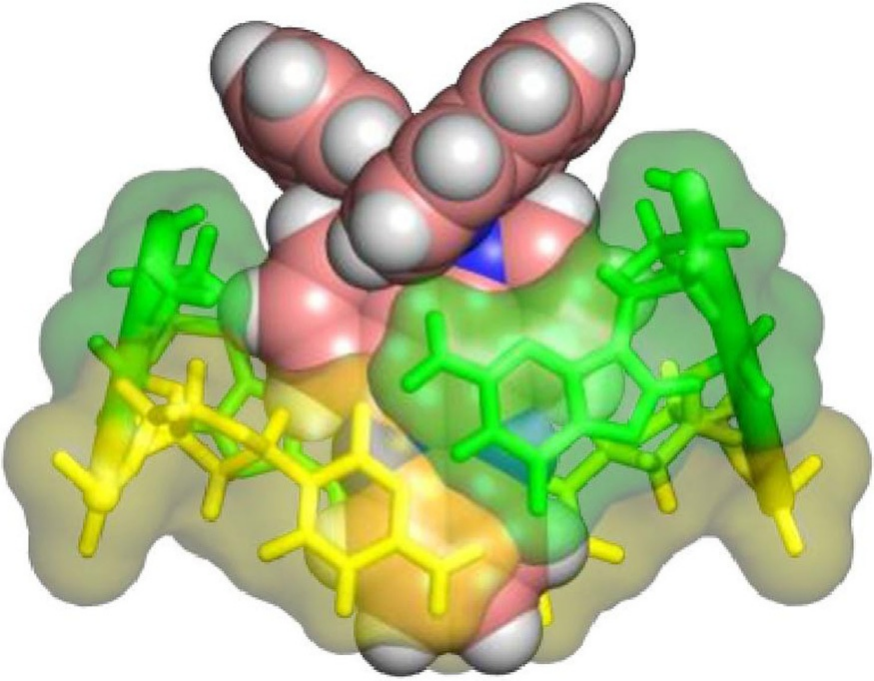
Hypothetical model of Λ‐[Ru(phen)_2_(dppz)]^2+^ intercalated symmetrically into a 5′‐CG‐3′ step. The DNA bases are coloured according to type, with guanine in green and cytosine in yellow. The complex is coloured with carbon in pink, nitrogen in blue and hydrogen in white. Please note that the dppz nitrogen atoms are under the DNA bases and partially inaccessible to solvent.

Furthermore, recent quantum mechanical calculations suggested that when intercalated into DNA, the emissive ^3^MLCT states located on the ancillary ligands are favoured in contrast to water, in which the dark ^3^MLCT and ^3^IL states located on the dppz group predominate.^18^ As a consequence of shallower intercalation at a 5′‐CG‐3′ step, the extent of π stacking between the dppz group and DNA bases, as well as the environment of the phen or bpy groups, would be affected both, which could be expected to contribute significantly to the differences in photophysical properties compared to at a 5′‐TA‐3′ step. This study suggests that the binding specificity of Λ‐Ru‐dppz complexes is far greater than previously considered and will therefore be a subject for further systematic study.

## Supporting information

As a service to our authors and readers, this journal provides supporting information supplied by the authors. Such materials are peer reviewed and may be re‐organized for online delivery, but are not copy‐edited or typeset. Technical support issues arising from supporting information (other than missing files) should be addressed to the authors.

Supplementary
