# Supplementary material for: Guanine Can Direct Binding Specificity of Ru–dipyridophenazine (dppz) Complexes to DNA through Steric Effects
Source: Chemistry. 2017 Feb 6;23(21):4981–5. doi: 10.1002/chem.201605508 (PMC5412927; doi:10.1002/chem.201605508)
Supplement: Supplementary file 1 — Supplementary [file CHEM-23-4981-s001.pdf]

CHEMISTRY
A European Journal

Supporting Information

Guanine Can Direct Binding Specificity of Ru–dipyridophenazine (dppz) Complexes to DNA through Steric Effects

James P. Hall,*[a, b] Sarah P. Gurung,[a, b] Jessica Henle,[a] Patrick Poidl,[a] Johanna Andersson,[c, d] Per Lincoln,[c] Graeme Winter,[b] Thomas Sorensen,[b] David J. Cardin,[a] John A. Brazier,[e] and Christine J. Cardin*[a]

chem_201605508_sm_miscellaneous_information.pdf

Materials and Methods

UV Melting Experiments

Chemicals were purchased from Sigma-Aldrich and oligonucleotides from Eurogentec. Oligonucleotides were purified by reverse-phase HPLC. UV spectroscopy was carried out using an Agilent Cary 100 with a temperature controlled 6 x 6 cell changer.

Oligonucleotides, at a final concentration of 3 µM duplex, were dissolved in 40 mM sodium cacodylate buffer at pH 7.0. Solutions were annealed, prior to running the melting experiments, by heating the samples 90°C and allowing them to slowly cool to room temperature. UV spectra were recorded at 260 nm at 1°C intervals between 20-90°C, with a temperature change rate of 1°C/min in a 1 cm pathlength quartz cuvette. Initially, attempts were made to study the 4-mer duplex that gave the crystal structure. However, as the theoretical melting temperature of this duplex in dilute solution is -21°C, no melting curve could be observed. We therefore utilised DNA 8-mers with sequence d((5-BrC)GG(5-BrC)G(5Br-C)(5-BrC)G) and d(CGGCGCCG), which were stable enough to enable us to perform the UV denaturation experiments.

Crystallization

The oligonucleotides, d((5Br-C)GGC) and d(GCCG) were purchased from ATDBio as solids purified by HPLC. Crystals containing the oligomers and complexes were grown using vapour diffusion from sitting drops at 18 °C. All three systems were crystallized from similar conditions. All drops contained 1 µL 1 mM duplex oligonucleotide and 6 µL of a solution containing 40mM Na-cacodylate pH 7, 12 mM spermine, 20 mM $BaCl_2$, 80mM KCl and 10% 2-methyl-2,4-pentanediol. These were equilibrated against 35% (v/v) 2-methyl-2,4-pentanediol for 2 weeks, after which orange hexagonal crystals grew.

X-ray Data Processing

The data were collected on beamline I02 at Diamond light source. Data collection, processing and refinement statistics are given in table S1. In all cases the data were processed using XDS[1] and XSCALE with xia2[2]. The structures were solved using the anomalous signal from both Ba^{2+} and Br with SHELXC/D/E[3] through the CCP4i[4] interface. All three structures were built using Coot[5], refined using REFMAC5[6] and deposited in the Protein Data Bank.

However, during refinement of structure 2, it became apparent that the diffraction from the crystal was anisotropic. This anisotropy was analysed using the UCLA diffraction anisotropy server[7] which found that the resolution limits, defined as the point at where the F/σF drops below 3, was 1.3 Å in the a* and b* directions but only 1.9 Å in the c* direction. Whilst this is classed as mild anisotropy, a number of artefacts were present in the difference map (Figure S1) which made proceeding with refinement challenging. The data underwent ellipsoidal truncation to give truncated structure factors. In this case, the model was refined against the truncated data. Both the original and truncated data have been deposited with the entry in the Protein Data Bank.

Table S1: Crystallographic Data Processing and Refinement Statistics for Three Structures Containing DNA-Bound Ru-dppz Complexes

Structure	1	2	3
Complex	$[Ru(bpy)_2(dppz)]^{2+}$	$[Ru(phen)_2(dppz)]^{2+}$	$[Ru(phen)_2(dppz\text{-}11,12\text{-}Me)]^{2+}$
**Data Collection**			
X-ray wavelength, Å	1.7712	0.8266	0.8266
Exposure time, s	0.1	0.1	0.1
Number of images	900	900	900
Wedge collected, °	90	90	90
Structure Solution Method	SAD	SAD	SAD
**Data Processing**			
Space group	$P\,6_4\,2\,2$	$P\,6_4\,2\,2$	$P\,6_4\,2\,2$
Unit cell *a*, *c*, Å	66.90, 36.02	67.34, 35.79	67.72, 35.85
Resolution, Å	21.90-1.85 (1.90-1.85)*	24.52-1.28 (1.31-1.28)	35.85-1.56 (1.60-1.56)
R_{merge}	0.069 (0.148)	0.044 (0.872)	0.061 (0.984)
R_{meas}	0.089 (0.248)	0.055 (0.983)	0.074 (1.089)
R_{pim}	0.031 (0.139)	0.018 (0.319)	0.025 (0.349)
Total number of observations	31338	117212	65849
Total number of unique observations	4297	12524	7169
I/σI	13.7 (2.1)	17.9 (2.2)	16.8 (2.3)
$CC_{1/2}$	0.998 (0.975)	0.999 (0.882)	0.999 (0.883)
Completeness, %	98.1 (86.4)	98.0 (98.0)	98.2 (98.6)
Multiplicity	7.3 (2.0)	9.4 (9.4)	9.2 (9.6)
Mid-slope of anom normal probability†	1.539	1.259	1.257
*Outer shell statistics shown in parentheses			
**Refinement**			
No. Reflections	3885	7986#	6787
R_{work}/R_{free}	0.17/0.19	0.14/0.17	0.14/0.19
**No. Atoms**			
DNA	159	159	159
Ligands	49	51	53
Water	53	23	49
**Average B-factors**			
DNA	36.5	26.07	28.26
Ligands	32.76	22.75	24.03
Water	47.66	33.67	46.96
**rmsd**			
Bond Lengths	0.011	0.01	0.02
Bond Angles	2.565	2.461	2.75
**PDB ID**	**5LFS**	**5LFW**	**5LFX**

#Data were ellipsoidally truncated during refinement, hence the discrepancy between the number of reflections obtained during data collection and those used for refinement. †A normal probability plot is one method of checking for anomalous signal in X-ray crystallographic data. A value of >1 may indicate that signal is present[8].

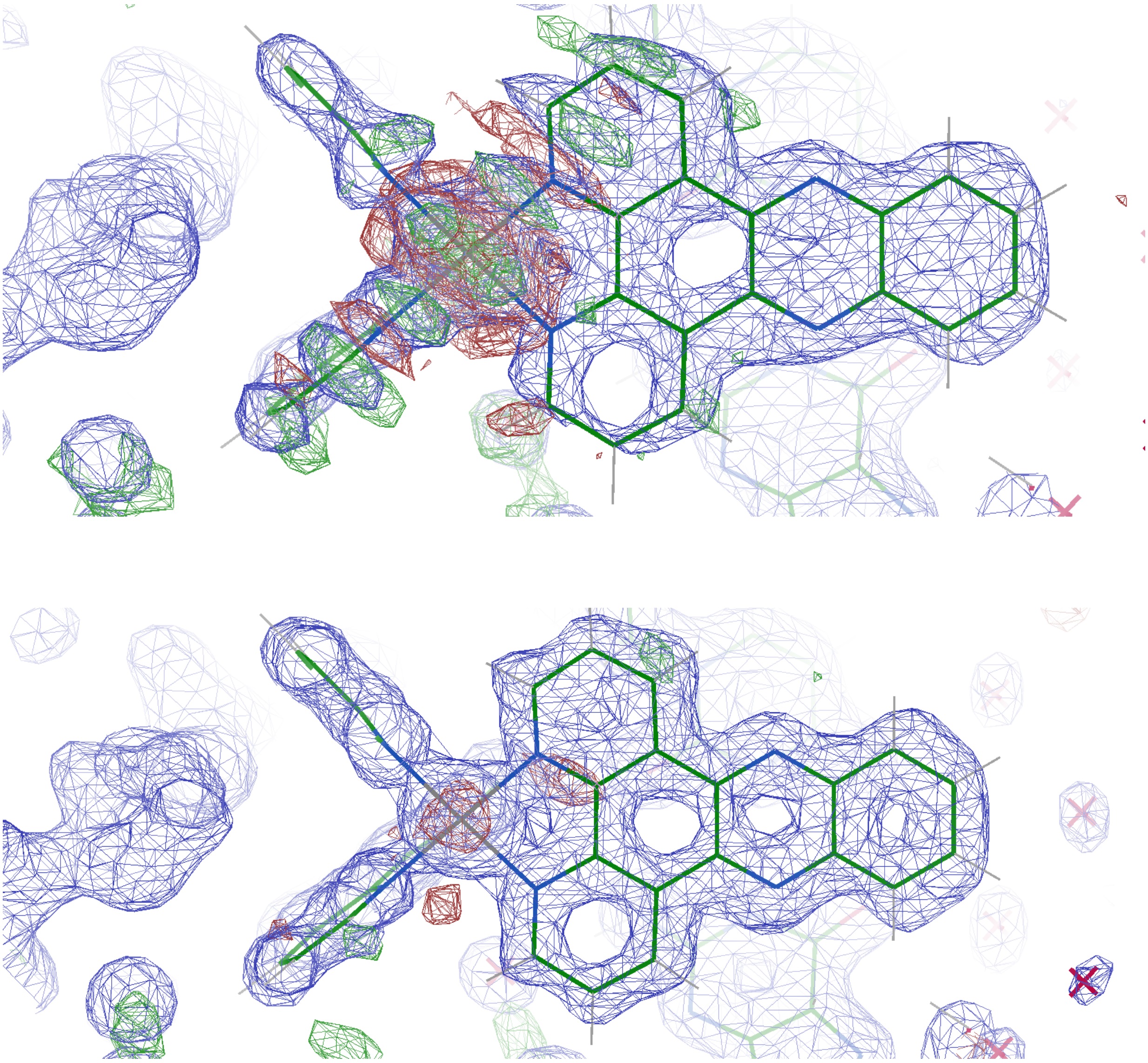

Figure S1: Quality of the $2F_o$-F_c electron density map (blue) and F_o-F_c difference map (positive in green, negative in red) before (top) and after (bottom) ellipsoidal truncation. Contour level of $2F_o$-F_c map is 0.5 e/Å^3 and F_o-F_c map is 0.3 e/Å^3. Atoms are coloured according to type, with carbon in green, ruthenium as a light grey cross, hydrogen in grey, nitrogen in blue and oxygen in red. Water molecules are drawn as red crosses.

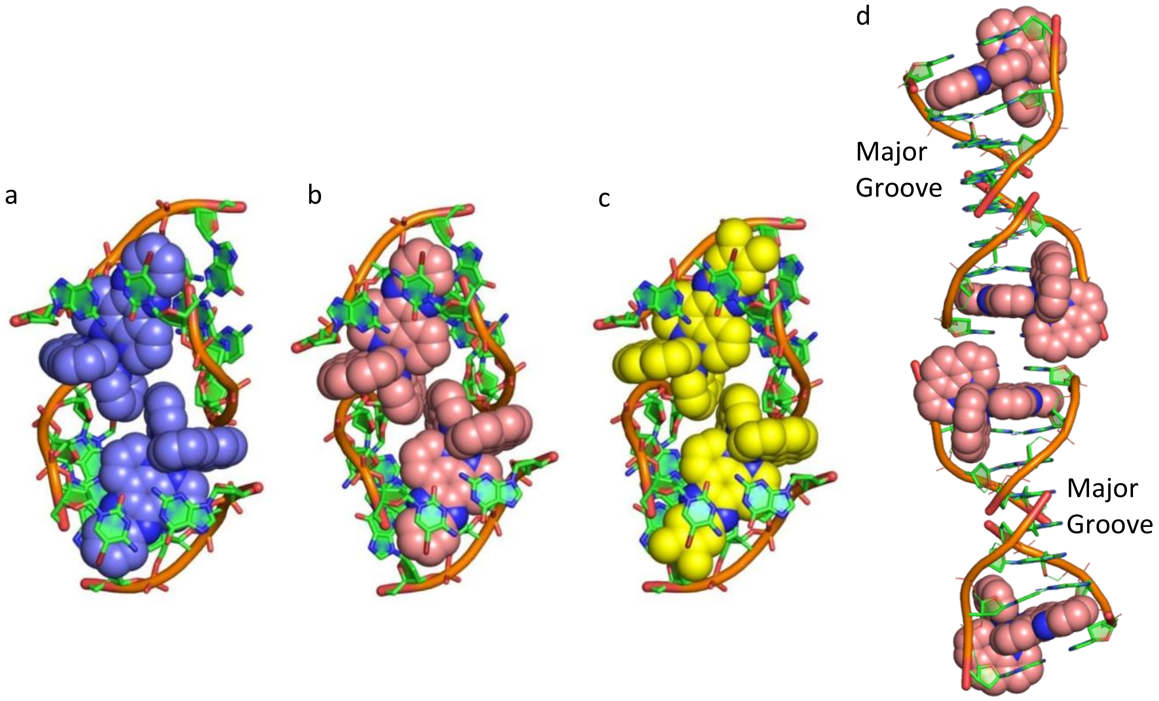

Figure S2: Crystal packing interactions in structures 1-3. (a) Λ-[Ru(bpy)$_2$(dppz)]$^{2+}$ (purple). (b) Λ-[Ru(phen)$_2$(dppz)]$^{2+}$ (pink). (c) Λ-[Ru(phen)$_2$(dppz-11,12-Me)]$^{2+}$. Note that two symmetry-related duplexes pack together so that the ancillary ligands of the complex come into close proximity with each other. (d) A view, into the minor groove, of four duplexes stacking on each other in structure (2). DNA atoms are coloured according to type with carbon in green, nitrogen in blue, phosphorus in orange, oxygen in red and bromine in brown.

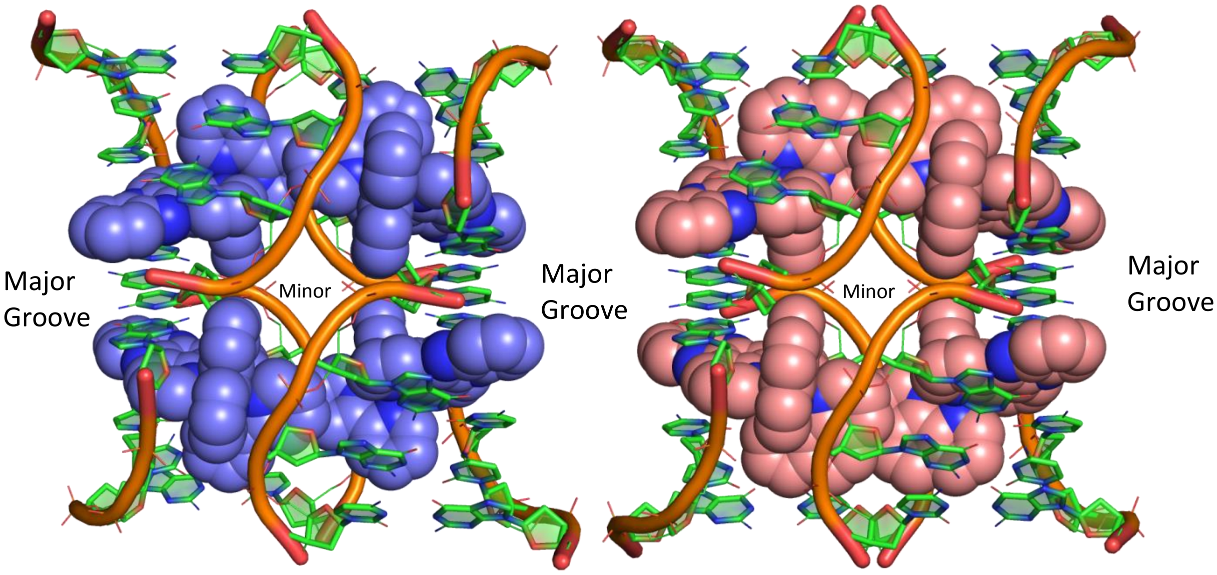

Figure S3: A comparison of the binding site in the structure (1) containing Λ-[Ru(bpy)$_2$(dppz)]$^{2+}$ (left) and structure (2) containing Λ-[Ru(phen)$_2$(dppz)]$^{2+}$ (right). DNA atoms are coloured according to type, with carbon in green, nitrogen in blue, phosphorus in orange and oxygen in red. “Minor” indicates the DNA minor groove.

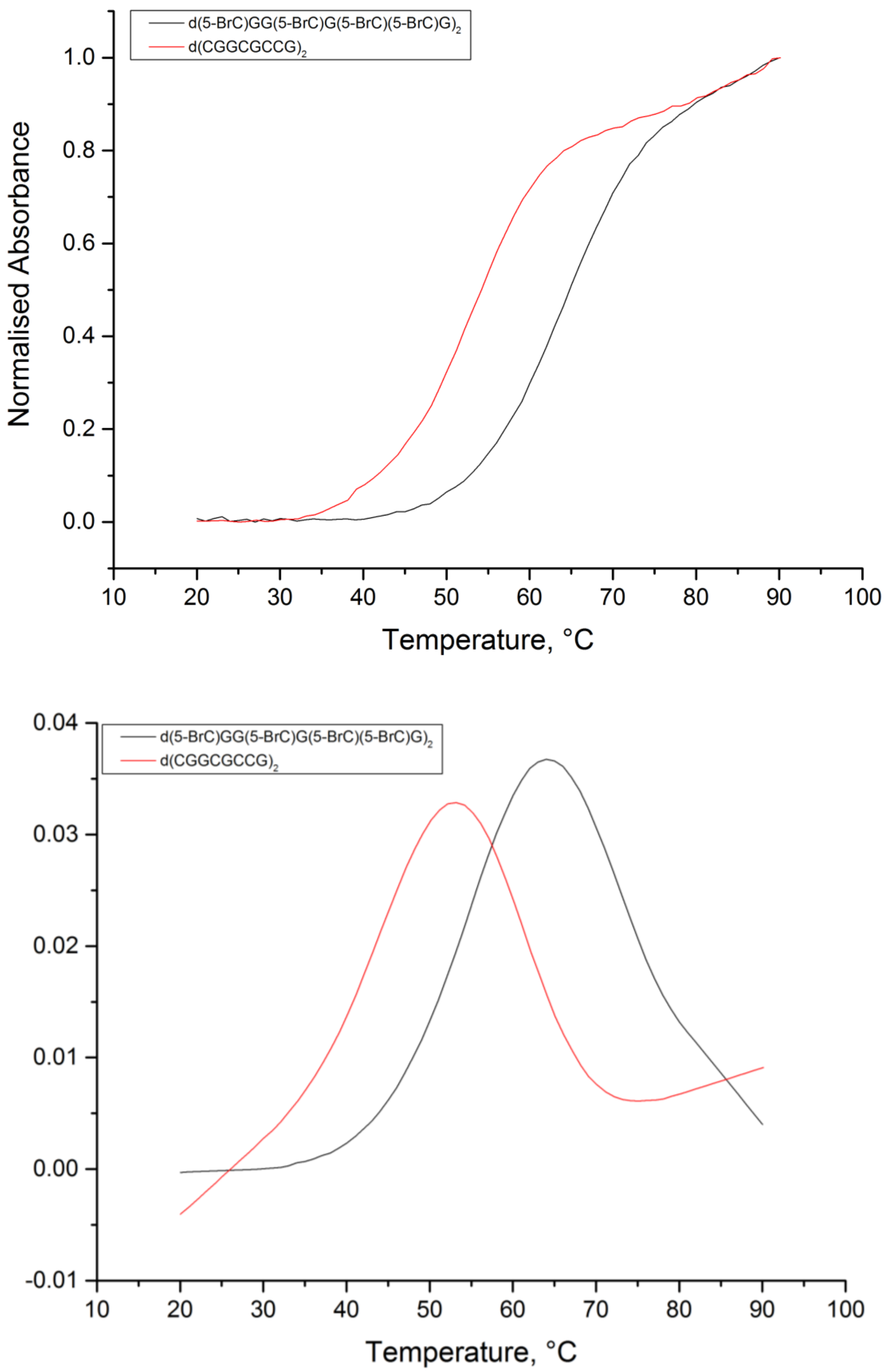

Figure S4: UV melting curves (top) and first derivatives for d(CGGCGCCG)$_2$ and d((5-BrC)GG(5-BrC)G(5-BrC)(5-BrC)G)$_2$. Note the positive shift of 11 °C in the melting temperature when all the cytosine bases are brominated.

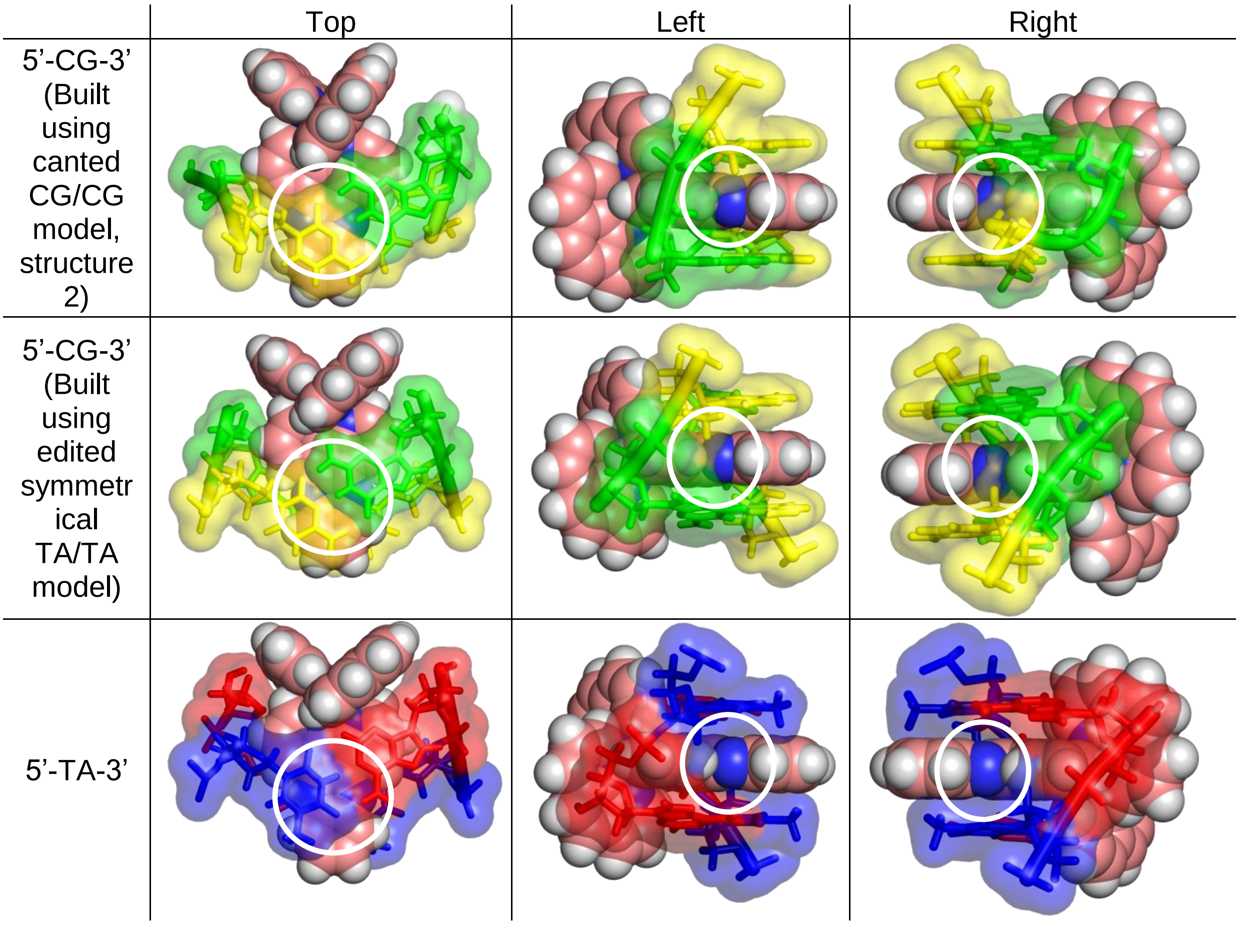

Figure S5: Three views of Λ-[Ru(phen)$_2$(dppz)]$^{2+}$ intercalated into two hypothetical 5'-CG-3' steps and a 5'-TA-3' site in a previously reported X-ray crystal structure[9]. Note that when intercalated into a 5'-CG-3' step, using either a canted or symmetrical binding site as a starting model, the dppz nitrogen atoms are partially occluded from the solvent. However, when intercalated into a 5'-TA-3' site both nitrogen atoms are accessible. The carbon atoms in the complex are coloured pink, with hydrogens in white and nitrogen in blue. The DNA bases are coloured according to type with A in red, T in blue, C in yellow and G in green. The dppz nitrogen atoms are ringed in white in each image.

References

[1] W. Kabsch, *Acta. Crystallogr.* **2010**, *D66*, 125–132.

[2] G. Winter, *J. Appl. Crystallogr.* **2009**, *43*, 186–190.

[3] G. M. Sheldrick, *Acta. Crystallogr.* **2008**, *A64*, 112–122.

[4] Collaborative Computational Project Number 4, *Acta. Crystallogr.* **1994**, *D50*, 760–763.

[5] P. Emsley, B. Lohkamp, W. G. Scott, K. Cowtan, *Acta. Crystallogr.* **2010**, *D66*, 486–501.

[6] G. N. Murshudov, A. A. Vagin, E. J. Dodson, *Acta. Crystallogr.* **1997**, *D53*, 240–255.

[7] M. Strong, M. R. Sawaya, S. Wang, M. Phillips, D. Cascio, D. Eisenberg, *Proc. Natl. Acad. Sci.* **2006**, *103*, 8060–8065.

[8] P. L. Howell, G. D. Smith, *J. Appl. Cryst.* **1992**, *25*, 81–86.

[9] H. Niyazi, J. P. Hall, K. O'Sullivan, G. Winter, T. Sorensen, J. M. Kelly, C. J. Cardin, *Nat. Chem.* **2012**, *4*, 621–628.
